# The epitranscriptome of high-grade gliomas: a promising therapeutic target with implications from the tumor microenvironment to endogenous retroviruses

**DOI:** 10.1186/s12967-023-04725-z

**Published:** 2023-12-09

**Authors:** Christian K. Ramsoomair, Michele Ceccarelli, John D. Heiss, Ashish H. Shah

**Affiliations:** 1 Section of Virology and Immunotherapy, Sylvester Comprehensive Cancer Center, University of Miami Miller School of Medicine, 1095 NW 14Th Terrace, Miami, FL 33136 USA; 2 Medical Scientist Training Program, University of Miami Miller School of Medicine, 1095 NW 14Th Terrace, Miami, FL 33136 USA; 3 Sylvester Comprehensive Cancer Center, Miller School of Medicine, University of Miami, 1550 N.W. 10Th Avenue, Miami, FL 33136 USA; 4 Surgical Neurology Branch, Disorders and Stroke, National Institute of Neurological, National Institutes of Health, Bethesda, MD 20892 USA

**Keywords:** Glioblastoma, Epitranscriptomics, RNA modifications, Endogenous retrovirus, Microenvironment

## Abstract

Glioblastoma (GBM) comprises 45.6% of all primary malignant brain cancers and is one of the most common and aggressive intracranial tumors in adults. Intratumoral heterogeneity with a wide range of proteomic, genetic, and epigenetic dysregulation contributes to treatment resistance and poor prognosis, thus demanding novel therapeutic approaches. To date, numerous clinical trials have been developed to target the proteome and epigenome of high-grade gliomas with promising results. However, studying RNA modifications, or RNA epitranscriptomics, is a new frontier within neuro-oncology. RNA epitranscriptomics was discovered in the 1970s, but in the last decade, the extent of modification of mRNA and various non-coding RNAs has emerged and been implicated in transposable element activation and many other oncogenic processes within the tumor microenvironment. This review provides background information and discusses the therapeutic potential of agents modulating epitranscriptomics in high-grade gliomas. A particular emphasis will be placed on how combination therapies that include immune agents targeting hERV-mediated viral mimicry could improve the treatment of GBM.

## Key points

- In vivo studies link epitranscriptomic dysregulation with glioma pathogenesis.
- TCGA shows elevated regulator expression is associated with increased mortality in GBM patients.
- RNA modifications negatively correlate with GBM immunogenicity and thus reduce the efficacy of immunotherapies in GBM patients.
- RNA modification regulators represent promising therapeutic agents for reducing mortality and immunotherapy adjuvants for enhancing tumor immunogenicity.

## Introduction

Glioblastoma (GBM) comprises 45.6% of all primary malignant brain cancers and is one of the most common and aggressive intracranial tumors in adults [1]. Due to its highly aggressive nature and inevitable recurrence, its disease course has one of the highest mortalities, with a 5-year survival rate of only 5% despite maximal surgical resection and adjuvant chemoradiation [2].

GBM is a highly heterogeneous neoplasm with a wide range of proteomic, genetic, and epigenetic dysregulation [3]. Many enzymes responsible for regulating protein and DNA modifications are currently targets of cancer trials and therapies. However, studying RNA modifications, or RNA epitranscriptomics, is a new frontier within neuro-oncology [4]. Simply put, epitranscriptional modifications include dynamic, covalent modifications that can affect the stability, translation, and function of RNA. Although eukaryotic RNA modifications have been known since the 1970s, most studies focused on transfer RNA (tRNA) and ribosomal RNA (rRNA). Only within the last decade has there been extensive characterization of modifications of mRNA and various non-coding RNAs. RNA modifications have steadily moved towards the limelight as scientists appreciated that RNA does not only act as an effector molecule (tRNA and rRNA) or intermediate in protein synthesis (mRNA) but also directly affects gene expression via non-coding RNAs such as microRNA (miRNA) and long ncRNA (lncRNA) [5–7].

Few dedicated studies of posttranscriptional modifications and their potential as therapeutic targets occurred initially. Still, in the past few years, this field has become an active field of investigation for brain tumors [4]. Several lines of evidence suggest that epitranscriptomic dysregulation contributes to glioma pathogenesis [4, 8–10]. The three major epitranscriptomic players implicated in both physiologic regulation and disease include “writers” that add a specific modification, “erasers” that remove a specific modification, and “readers” that identify and bind modified nucleotides.

A common consequence of pretranscriptional (i.e., epigenetics) and posttranscriptional (i.e., epitranscriptomic) dysregulation is the activation of transposable elements (TEs). Interestingly, in the last decade of cancer research, a form of TEs known as endogenous retroviruses (ERVs) has been shown to play a dichotomous role in driving oncogenesis and serving as potential antigens for immunotherapy [11]. First described in 1981, human ERVs (hERVs) are relics of retroviral infection of the ancestral germline. hERV genes account for nearly 9% of the human genome and are transcriptionally silent in normal cells. hERV expression is associated with many cancers. hERV expression also is related to autoimmune and neurodegenerative diseases. Augmenting hERV inflammatory effects through a cellular state known as “viral mimicry” is being explored to sensitize tumors to immunotherapy [12]. Viral mimicry enhances hERV expression by reversing silencing marks that suppress hERV gene expression. The fundamental premise is that a transient increase in hERV expression may induce an innate antiviral and adaptive immune response that sensitizes and homogenizes tumor cells for immunotherapy.

Recent publications extensively reviewed how RNA modifications participate in cancer and glioma development/progression, regulate the tumor microenvironment (TME), and encourage the development of drug resistance [4, 9]. This review aims to provide background information about epitranscriptomics and discuss the therapeutic potential of modulating epitranscriptomics in high-grade gliomas and GBM. We will also further discuss the rationale for using a hERV-mediated viral mimicry strategy as part of a combination immunotherapy for GBM.

### Aberrant RNA modifications in GBM

**Fig. 1:**
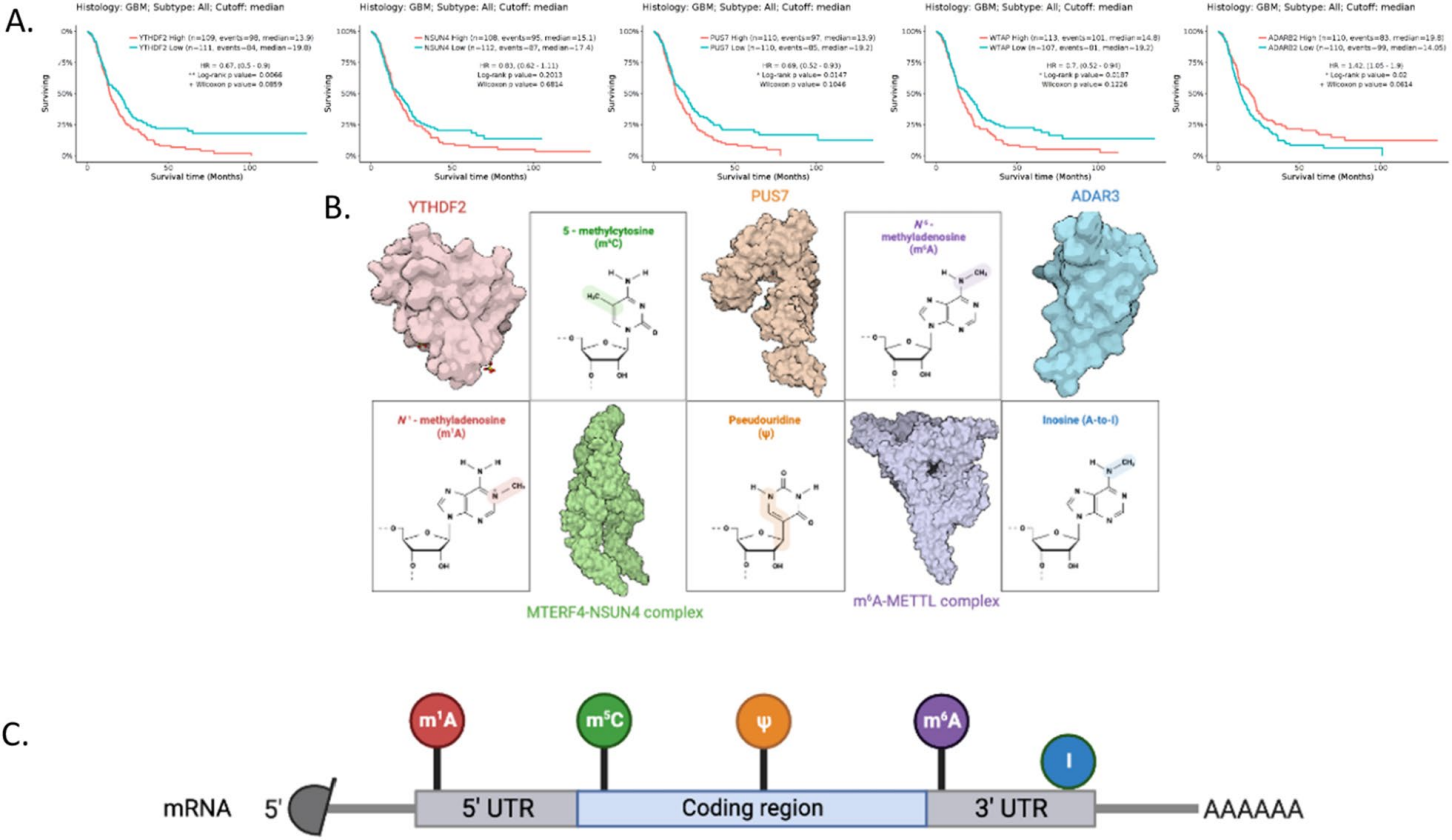
Select RNA modification regulators and glioma survival. **A** Kaplan–Meier curves shown for RNA **B** modification regulators YTHDF2, NSUN4, PUS7, WTAP, and ADAR3. Note both IDH wildtype and mutant status are included. GlioVis data portal for visualization and analysis of brain tumor expression datasets [123]. **B** PDB images of each protein or protein complex [131] is shown adjacent to the respective modification involved. Of note, YTHDF2 has been shown to be a reader molecule for both m1A and m6A [124, 125]. **C** mRNA modifications are shown in the most probable area based on its respective transcriptome-wide distribution [8]. CDS, coding sequence; UTR, untranslated region

In the context of glioma pathogenesis and progression, the most relevant RNA modifications are N6-methyladenosine (m6A), 5-methylcytidine (m5C), N1-methyladenosine (m1A), hydroxymethylcytidine (hm5C), pseudouridine (Ψ), and adenosine-to-inosine (A-to-I) RNA editing [9]. Elevated transcript levels of RNA modification writers/readers have been associated with increased mortality in GBM patients, as illustrated in Fig. 1. One notable exception is ADAR3, whose transcript levels have oddly been found to be inversely correlated to its protein expression [13].

**Fig. 2:**
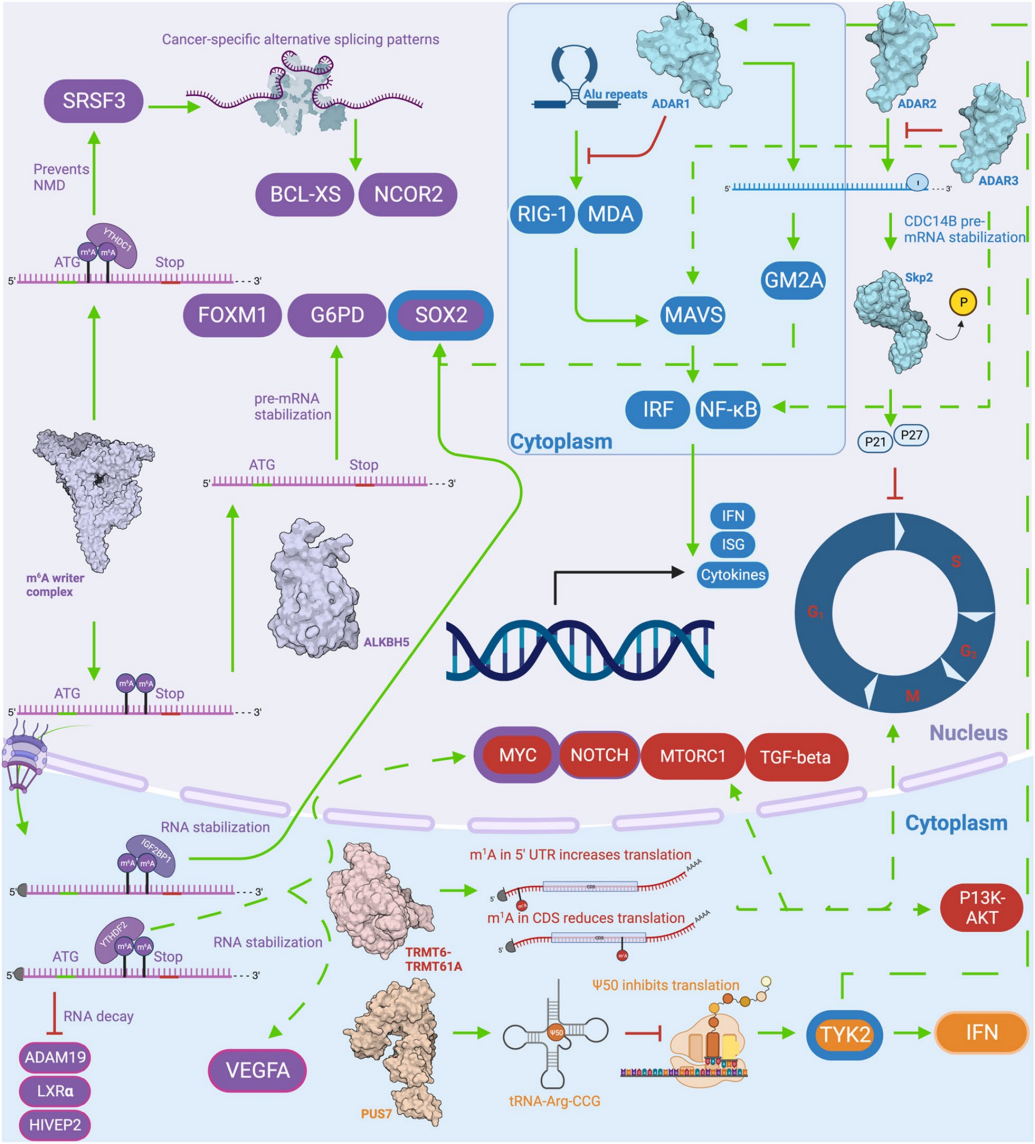
major Oncogenic Pathways in Glioma Epitranscriptome. Following the color scheme established in Fig. 1, major oncogenic pathways proceeding through the m6A writer complex, m6A eraser ALKBH5, A-to-I RNA editors ADAR1/2/3, m1A writer TRMT6-TRMT61A, and pseudouridine writer PUS7 are illustrated

The following subsections will review the physiologic role of each RNA modification on gene regulators. Furthermore, Table 1 summarizes the findings of dysfunction associated with each modification in glioma, and Fig. 2 illustrates major pathways where mechanistic studies have been completed.

**Table 1:** Dysfunction associated with RNA modifications in glioma

RNA modification	Regulator	Target genes/pathways	Effect on glioma development	Sources
m6A	YTHDC1	*Prevents* NMD of arginine rich SRSF3 transcripts	Promotes proliferation and progression in primary cells	[109]
	YTHDF2	*Stabilized by* EGFR/SRC/ERK pathway *Facilitates* mRNA decay of LXRα and HIVEP2 *Stabilizes MYC* and *VEGFA* transcripts	Promotes proliferation, invasion, and tumorigenesis in GSCs	[110, 111]
	HNRNPA2B1	*Activates* AKT and STAT3 signaling pathway *Enhances* expression of *Bcl-2* and *PCNA*	Promotes proliferation and inhibits apoptosis in cell line and xenograft mouse	[112]
	METTL3	*Modulates* Bcl-2 and SRSF protein expression to regulate apoptosis	Promotes proliferation and progression in cell line, primary cells, and xenograft mice	[109, 113, 114]
	ALKBH5	*Stabilizes* G6PD *Activates* pentose phosphate pathway *Enhances* SOX2 and FOXM1 expression	Promotes proliferation in cell lines	[109, 115–117]
	FTO	*Inhibited* by 2-HG secondary to IDH1 mutations *Stabilizes* c-Myc and CEBPA *Decreases* VPS25 expression	Context dependent. Tumorigenic role both when inhibited (hyper-m^6^A-methylation) and not with growth factor stabilization. Reduces glioma growth by increased glioma apoptosis	[109, 118, 119]
m1A	TRMT6	*Regulates* cell cycle, PI3K-AKT, TGF-beta, MTORC1, NOTCH, and MYC pathway	Promotes proliferation, migration, and invasion in rat and human cell lines	[120, 121]
	TRMT61A	*Targeted by* HIF1A target *Suppressed by* c-Myc inhibition (hypoxic conditions)	Promotes proliferation in rat cell line	[120, 122]
m5C	NOP2 NSUN4/5/7	–	Worsens prognosis	[123]
	NSUN6	–	Improves prognosis	[123]
A-to-I RNA Editing	ADAR1/ADAR	*Prevents* aberrant RLR activation *Prevents* PKR activation *Edits* GM2A	Promotes GSC proliferation and self-renewal in primary cells and xenograft mice Negatively associated with grade	[108, 124, 125]
	ADAR2/ADARB1	*Edits* GRIA2 *Edits* CDC14B pre-mRNA *Reduces* Skp2/p21/p27 cell cycle pathway	Inhibits proliferation and migration in cell lines Negatively associated with grade	[29, 124, 126]
	ADAR3/ADARB2	*Inhibits* (competitively) ADAR1/2 *Promotes* NF-κB activation	Promotes proliferation and progression in cell lines	[39, 124, 126, 127]
*ψ*	PUS7	*Modulates* TYK2–STAT1 pathway	Promotes proliferation and progression in GSCs and xenograft mice	[107, 128, 129]
	DKC1	*Upregulates* HIF1A, N-cadherin, and MMP-2	Inhibits proliferation, migration, and invasion in cell lines	[130]

*NMD* nonsense-mediated RNA decay, *SRSF3*, splicing factor 3, *G6PD* glucose-6-phosphate dehydrogenase, *2-HG* 2-hydroxy-glutarate, *IDH1* isocitrate dehydrogenase 1, *CEBPA* CCAAT enhancer binding protein alpha, *PKR*, protein kinase A, *MAVS* mitochondrial antiviral signaling protein, *RLRs* RIG-I-like receptors, *GRIA2* glutamate receptor ionotropic, AMPA, *CDC14B* cell division cycle 14B, *Skp2 S*-phase kinase associated protein 2, *TYK2–STAT1* tyrosine kinase 2-signal transducer and activator of transcription 1

#### M6A

Methylation of the adenosine at the N6 position (m6A) is the most common modification of mRNA and is primarily found in the 3’ untranslated region (UTR) [14]. The writers that catalyze this modification include methyltransferase enzymes methyltransferase-like 3 (METTL3), METTL14, and Wilms tumor 1-associated protein (WTAP) [14]. Examples of erasers that remove them are fat mass and obesity-associated protein (FTO) and alkB homolog 5 (ALKBH5) [15]. Readers involved in the m6A physiologic functions usually comprise the YT521-B homology (YTH) domain family of proteins or the heterogeneous nuclear ribonucleoprotein (HNRNP) proteins [9].

The physiologic function of m6A modifications involves pre-mRNA splicing, mature mRNA transport, and translation [9, 16, 17]. The HNRNPC reader regulates mRNA splicing, while the YTHDC1 reader regulates mature mRNA export [18, 19]. The YTHDF2 and YTHDF3 readers work on translation to accelerate and increase efficiency, respectively [20]. The METLL3 writer is involved in mature mRNA export and translation regulation [19]. Regarding m6A erasers, FTO and ALKBH5 both function as splicing-related erasers, with the latter also shown to enhance cellular stability [17, 21].

#### M1A

Adenosine methylation at the N1 position (m1A) is frequently found in the 5’ UTR of mRNA and tRNA. The tRNA methyltransferase 6/61A (TRMT6/61A) and tRNA methyltransferase 10C (TRMT10C) writers and YTHDF1/2/3 and YTHDC1 readers function to influence mRNA structural stability and increase translation [9, 22]. Additionally, m1A methylation may negatively impact the translation of coding sequences (CDS) in mitochondrial mRNA (mt-mRNA) [22]. AlkB homologs 1 and 3 (ALKBH1/3) act as erasers.

#### M5C

In addition to being found on DNA, the methylation of cytosine at the N5 position (m5C) is frequently found in the UTRs of mRNA [23]. Writers include NOL1/NOP2/SUN domain (NSUN) proteins and the DNA methyltransferase (DNMT) DNMT2 [9]. Only NSUN2 is known to be capable of modifying mRNA, while the rest of the writers solely act on rRNAs and tRNAs. Aly/REF export factor (ALYREF) serves as a reader, participating in transcript transport and translation efficiency regulation [24]. Interestingly, evidence has shown that m5C methylation of 5’UTRs and CDS may limit or altogether abolish translation, respectively, while modification of 3’UTRs may enhance expression [9]. m5C erasers have yet to be identified. Furthermore, m5C can be oxidized into hm5C by the Tet-family enzymes [25]. This most often occurs in coding regions and appears associated with translational activation.

#### Ψ

Isomerization of uracil (U) yields the pseudouridine (Ψ) modification. This is thought to be the most abundant RNA modification, mainly accumulating in rRNAs and tRNAs [26]. An RNA-dependent pseudouridine synthase (PUS) such as PUS1/3/7 in humans and centromere-binding factor 5 (Cbf5) in yeast usually catalyze this modification [27, 28]. Despite limited investigations on the Ψ modification in biological mRNAs, its ability to prevent pre-mRNA splicing, maintain mRNA stability, and increase mRNA translation is thought to result in improper protein expression [9].

##### A-to-I editing

Nucleotide base editing of adenosine to inosine is catalyzed by adenosine deaminases (ADARs) [29]. In addition to changing the primary sequence, it appears to affect mRNA native secondary structures [30]. In humans, there are three ADAR proteins: ADAR1 (ADAR), ADAR2 (ADARB1), and ADAR3 (ADARB2) [31]. While ADAR1 and ADAR2 are ubiquitously expressed and catalyze A-to-I RNA editing in many organs and at millions of sites in the human transcriptome [32, 33], ADAR3 expression is restricted to the brain and appears catalytically inactive [34–38]. Interestingly, in addition to dsRNA binding activity, ADAR3 has been shown to bind single-stranded RNA (ssRNA) in vitro [35]. Although not yet fully elucidated, ADAR3 appears to play a role in competitive inhibition of the catalytically active ADARs [39]. Adenosine deaminases are also known to act on tRNAs (ADATs) to catalyze A-to-I editing [40]. These enzymes remain understudied and incompletely characterized, with only 7 related papers on PubMed.

## RNA modification patterns in glioma

The role of RNA modification in cancers is incredibly complex and challenging to study, primarily due to limitations in sequencing technologies. Conventional sequencing techniques such as bulk RNA-seq and scRNA-seq quantify and normalize for mRNA expression but are not specifically developed to detect RNA modifications. Taguchi et al. recently reviewed developments in epitranscriptome spatial detection and data analysis [41]. Advancements in de novo sequencing will be discussed later in this review.

RNA modifications, namely m6A methylations, have been found to influence the tumor microenvironment (TME), immune cell-infiltrating characteristics, and, ultimately, patient outcomes [4, 42]. RNA modification patterns are particularly clinically relevant since they may portend responses to immunotherapy [42]. Similarly, Lin et al. utilized the TCGA-GBM and TCGA-LGG databases to explore patterns of differentially expressed m6A regulators in glioma [43]. Using principal component analysis, two glioma subgroups were identified, and one cluster was found to have a worse prognosis, higher WHO classification grade, and higher immune infiltration. Based on the combination of m6A RNA methylation and the landscape of the immune microenvironment, a novel 4-gene (TAGLN2, PDPN, TIMP1, EMP3) prognostic model was developed and validated with a good prediction. In addition, PDPN and TIMP1 were found to be highly expressed in high-grade glioma via The Human Protein Atlas database, and both correlated with m6A and macrophage marker CD68 in glioma tissue samples, which may serve as potential biomarkers for glioma prognosis.

Molecular alterations characterizations such as isocitrate dehydrogenase IDH1/2 mutations, 1p/19q codeletion, and MGMT promoter methylation, have improved the accuracy of diagnostics, prognostics, and prediction of treatment response for glioma patients. However, we have yet to observe any tangible improvement in either clinical management or patient outcomes. A potential utility of epitranscriptomics is to be used in conjunction with known glioma sub-entities to further advance tumor stratification and help guide treatment selection [15]. According to studies mining the Chinese Glioma Genome Atlas (CGGA), The Cancer Genome Atlas (TCGA), and Repository for Molecular Brain Neoplasia Data (REMBRANDT), epitranscriptomic regulators appear to be differentially expressed based upon molecular alterations and clinical attributes. For example, METTL3, FTO, and YTHDC1 are significantly differentially expressed between IDH-mutant and IDH-wildtype high grade gliomas [44]. Additionally, m6A writer METTL3 correlates with poor OS in IDH-wildtype but not in IDH-mutant gliomas [45].

A critical limiting factor of most epitranscriptomic cancer studies has been the scientific indifference towards RNA modifications other than m6A. Given the integrated process of interactions between different RNA methylation modifications, studying a single type of RNA modification in tumors may not comprehensively elucidate epitranscriptomic effects. A recent study by Li and colleagues recognized this. It developed a GBM score’ based on the differentially expressed genes (DEGs) between groups showing RNA modification patterns of writers that catalyze m1A methylation, m6A methylation, APA, and A-to-I RNA editing [46]. Out of the 26 writers analyzed, 15 were found to be more highly expressed in tumor tissue than adjacent normal tissue. Using Spearman’s correlation analysis, positive correlations occurred among several writers, which indicate that crosstalk among RNA modification writers may mediate the formation of distinct RNA modification patterns and GBM progression. After consensus clustering analysis, two clusters emerged, with one having a significantly shorter survival. In this cluster with higher mortality, GSVA was used to estimate the signaling pathways with gene set enrichment. Signaling pathways found to be significantly enriched were those involved in NK cell-mediated cytotoxicity, Toll-like receptor activation, JAK-STAT, and chemokine signaling. Additionally, using CIBERSORT, this same cluster was found to have higher levels of immunosuppressive cells such as M2 macrophages. Of note, several RNA modification writers, including RBM15, RBM15B, TRMT6, CLTP1, PABPN1, ADARB1, and CPSF1, were found to be positively associated with M0 macrophage differentiation.

The immunosuppressive tumor microenvironment has been identified as the primary reason for the failure of immune therapy in GBM patients; currently, there are no FDA-approved immunotherapies for GBM [47]. Accumulating evidence has shown a strong correlation between RNA modifications, namely m6A, and immunomodulation, and thus the efficacy of immunotherapies in GBM patients [43, 46, 48–50]. This correlation does not appear to be unique to primary brain tumors, as similar data has been found in colorectal cancer [51], colon cancer [52], gastric cancer [42], bladder cancer [53], lung adenocarcinoma [54], and head and neck squamous cell carcinoma [55]. This collection of recent investigations indicates that RNA modification shapes the complexity of the TME and its response to immunotherapy. Han et al. provide a more complete review of this field [56]. While exciting, extensive work is needed to understand modifications other than m6A, crosstalk between modifications, and cell types modulated by RNA modification. To fully understand the underlying mechanisms and therapeutic potential, further in vitro and in vivo studies are needed using meticulous methods to dissect RNA modifications and their regulators in tumor cells versus immune cells [56].

## Advancements in RNA modification de novo sequencing

Epitranscriptomic sequencing technologies may be divided into four fundamental approaches: antibody-based, reverse-transcription signature-dependent, enzyme-dependent, and chemically assisted [8]. Antibody-based methods are the most widely used modality for m6A, m5C, ac4C, and m7G modifications; however, drawbacks include limited specificity, lack of stochiometric outputs, and high input material requirements. Reverse transcription signature-dependent techniques rely on insertion-deletion mutations (indels), mismatches, and truncations at the modification sites. Still, they are prone to false positives due to errors in library preparation and sequencing or single-nucleotide polymorphisms [57, 58]. Enzyme-dependent sequencing technologies rely on various enzymes such as demethylases, endonucleases, and exonucleases to discriminate regular bases from modified ones [8]. The efficiency and sequence biases of the respective enzyme characterize its limitations. Lastly, chemical-assisted technologies combine chemical treatments with next-generation sequencing [8]. Recent chemical methods have addressed the gap between the dynamics and reversibility of RNA modifications by allowing absolute quantification at single-base resolution for m6A, pseudouridine, m5C, and ac4C.

Novel sequencing technologies that allow simultaneous detection of multiple modifications are vital to understand the crosstalk between modifications further and drive the field of epitranscriptomics. Third-generation methods such as SMRT and Nanopore sequencing hold promise but have faced challenges, including limited signal-to-noise ratios, complex algorithms, and high error rates and costs [59]. In addition, spatial epitranscriptomic elucidation of the mRNA modification profiles of individual cells in both spatial and temporal dimensions is likely to become an important area in developmental and cancer biology [8, 60].

## (h)ERVs, post-transcriptional regulation, and GBM

As occurs in pluripotent embryos, many cancers are known to reactivate transposable elements (TE) through chromatin remodeling, DNA hypomethylation, and histone modifications [12]. TEs are fundamentally heterogeneous, comprising two main types: DNA transposons and retrotransposons, which include Long Interspersed Nuclear Elements (LINEs), Short Interspersed Nuclear Elements (SINEs), and Long Terminal Repeat (LTR) Retrotransposons. Our focus in this review will be on LTR retrotransposons or human ERVs (hERVs).

The genome of an intact hERV provirus comprises at least 5’ and 3’ long terminal repeats (LTRs) flanking an internal *Gag* (group-specific antigen)*-Pro* (protease)*-Pol* (polymerase) polyprotein-coding sequence. *Gag* is cleaved by *Pro* to generate a virus-like particle that contains the fusion protein and ERV mRNA. hERVs may also contain a remnant envelope (*Env*) and other accessory genes but are generally not infectious [12]. HERV-K (HML-2), first reported in 1986, is the most recent hERV integrated into genomic DNA. Unlike other hERVs, HERV-K contains a near-full-length transcript in the human genome that includes open reading frames (ORFs) of HERV-K *Gag*, *Pol*, and *Env*, which can be read and translated into functional retroviral proteins [61].

While necessary for mammalian embryologic development, ERV activation and transposition can compromise host health secondary to aberrant transcriptional regulation; thus, constant surveillance is needed to maintain homeostasis [62]. Although the ERV regulatory network mechanisms are poorly understood, recent advancements have helped elucidate how mammals effectively regulate ERV expression.

ERV regulatory networks fundamentally consist of pre-transcriptional and post-transcriptional regulatory networks [63]. Pre-transcriptional regulators include zinc-finger proteins (ZFPs), TRIM28-SETDB1, human silencing hub (HUSH) complex, SWItch-sucrose non-fermentable (SWI-SNF) complex, MORC proteins, Lymphoid-specific helicase (LSH), and P-element-induced wimpy testis (PIWI)-interacting RNAs (piRNAs). Alternatively, piRNAs, nuclear exosome targeting (NEXT) complex, and RNA methylation comprise the post-transcriptional modulators. Table 2 summarizes the proteins and function of the ERV expression regulators.

**Table 2:** Known Regulators of hERVs

Pre-transcriptional	Notable Proteins involved	Function	Sources
ZFPs & TRIM28-SETDB1	*Recruits* HIRA complex, heterodimeric protein complex of DAXX, and ATRX	Binds to and methylates proximal DNA and trimethylates proximal H3K9 residues to silence the transcription of ZFP-bound ERV loci Replace canonical H3 proteins with the transcriptionally repressive non-canonical H3.3 variant	[132] [133]
HUSH Complex	*Composed of* three protein subunits, MPP8, PPHLN1, and TASOR *Recruits* MORC2	Universally represses TE transposition and expression by performing targeted repression of loci corresponding to intron-less RNA	[134–137]
SWI-SNF Complex	*Composed of* PBRM1	Rearrange location of histone proteins in chromatin, which impacts loci accessibility for epigenetic modification	[138]
MORC	*Facilitates* HUSH, DNA methylators, and DAXX-ATRX	Compacts chromatin through multimeric assemblies that trap DNA loops to restrict access, which influences gene expression	[139]
LSH	*Facilitates* DNMT enzymes and H3K9 trimethylating enzymes	Permits accessibility of DNA transcription factors and chromatin-modifying enzymes to regulate ERV expression	[140, 141]
PIWI-interacting RNAs	*Utilizes* argonaute proteins *Guides* H3K9 tri-methylating and DNA methylating enzymes	Guides DNA and histone silencing activity proximal to loci complementary to the piRNA guide	[142]
Post-transcriptional			
PIWI-interacting RNAs	*Utilizes* argonaute proteins	Recognize and cleave complementary ERV mRNAs	[142]
NEXT Complex	*Composed of* RBM7, ZCCHC8, and MTR4 *Recruited by* HUSH	Degrades intron-less RNA	[143, 144]
m6A RNA Methylation	*Deposited by* METTL3-METTL14 *Recognized by* YTHDFs	m6A on 5'UTR signals for ERV mRNA degradation	[77]

*HIRA* histone regulator A, *DAXX* death-domain-associated protein, *ATRX* ATP-dependent helicase, *MPP8* methyl-H3K9-binding protein *PPHLN1* periphilin 1, *TASOR* transcription activation suppressor, *MORC2* microrchidia 2, *PBRM1* Polybromo 1, *DNMT* DNA methyltransferase, *RBM7* RNA-binding motif protein 7, *ZCCHC8* zinc-finger CCHC-type containing 8, *MTR4* ATP-dependent RNA helicase DOB1

### hERV-mediated Oncogenesis

hERVs may drive oncogenesis in two main ways: (1) indirect transcriptional regulation of oncogenes/tumor suppressors and (2) expression of oncogenic HERV proteins [11].

There is accumulating support for hERVs as an oncogenic driver and emerging target for treatment via epigenetic silencing. On the other hand, the role of post-transcriptional silencing and RNA modifications of hERV transcripts in glioma remains virtually unexplored. This subsection briefly recounts major hERV-mediated oncogenic findings and provides context for the onco-exaptation discussion later in the review.

Akin to its role in development, hERVs may serve as alternative promoters for proximal genes in malignant cells and cryptic transcription start sites to produce aberrant CDS mRNA [12]. Similarly, onco-exaptation may proceed by hERVs acting as enhancers [64]. Moreover, HERV-K-derived sequences appear to interrupt and inactivate tumor suppressor BRCA2 and DNA repair gene XRCC1 in glioma cells [65].

Through c-MYC proto-oncogene activation, HERV-K *Env* and accessory proteins Rec and Np9 have been linked to tumorigenesis in various cancers [66]. Rec and Np9 have been proposed to bind to the transcriptional repressor promyelocytic leukemia zinc finger (PLZF), which mediates the expression of the protooncogene c-MYC and suppressor genes p53 and p21 [67]. Np9 has also been associated with amplifying Notch signaling via binding to and initiating Ligand of Numb Protein X (LNX) degradation [68]. Interestingly, in glioma cells, LNX protein has been found to be decreased [69]. Therefore, the HERV-K Rec and Np9 represent putative oncogenes and require further investigation as therapeutic targets in gliomas displaying signs of hERV activation.

Finally, hERVs have been proposed to be responsible for the stem-cell phenotype in cancer stem cells (CSCs) [11, 61]. CD133, a common glioma stem-cell marker, strongly correlates with hERV expression in melanoma cell lines. Treatment with reverse transcriptase inhibitors lowers HERV-K expression and CD133 + melanoma cell populations [70]. Moreover, the HERV-K env is overexpressed in pluripotent stem cells (PSC) but downregulated during neuronal differentiation. This same protein interacts with CD98HC, activates the mTOR pathway, and induces epigenetic changes through lysophosphatidylcholine acyltransferase (LPCAT1) [71]. CD98 is widely expressed in astrocytic tumors, where it has been suggested to promote oncogenic transformation by facilitating amino acid transport [72].

### Epigenetic and m6A epitranscriptomic crosstalk

Chemical modifications on DNA, RNA, and proteins (e.g., histones) impact gene regulation. As discussed above, installing an RNA m6A modification alters mRNA stability and translation. Furthermore, emerging data suggests that m6A methylation influences physiological regulation beyond post-transcriptional mechanisms [73]. While loss of m6A writers and nuclear m6A readers are known to be developmentally lethal, knockout of the YTHDF family, cytoplasmic m6A readers involved in transcript decay, has not been shown to recapitulate lethality [74, 75]. Although alternative explanations, such as compensation from other readers, are undoubtedly reasonable, evidence that suggests m6A methylation feeds back onto epigenetic circuits has rapidly accumulated.

A vital function of heterochromatin is restraining the activity of embedded satellite repeats and transposable elements [76]. Endogenous retroviruses (ERVs) are a prominent class of retrotransposons that necessitate constitutive silencing by regulation machineries, traditionally understood to comprise epigenetic processes. Remarkably, three separate research groups have recently reported a role for m6A in regulating ERVs through an element known as intracisternal A particle (IAP) in mouse embryonic stem cells (mESCs) [77–79]. m6A on the 5′UTR of the IAP mRNA recruits YTHDF readers for mRNA degradation. Thus, m6A levels are inversely correlated with mRNA and protein levels of IAP. Knockout of Mettl3 writers and rescue by a catalytically inactive form failed to restore H3K9me3 levels at IAP elements, while knockout of Alkbh5 erasers significantly increased H3K9me3 levels at these sites. Thus, this data suggests a positive link between m6A deposition and levels of H3K9me3, a significant molecular feature of heterochromatin.

The current explanation for these findings is that m6A RNA modifications catalyzed by Mettl3 are known to interact with Ythdc1 readers. This interaction, confirmed via chromatin immunoprecipitation followed by sequencing (ChIP-seq) with enrichment at H3K9me3-rich transposable elements, has a role in mediating retrotransposon silencing and maintaining mESC identity [79]. In addition, Ythdc1 appears to guide Mettl3 and facilitate its interaction with chromatin, tripartite motif containing 28 (TRIM28), and SET-domain-bifurcated histone lysine methyltransferase 1 (SETDB1). This aptly named m6A methyltransferase complex regulates H3K9me3 deposition at IAPs.

Despite some inconsistencies in the proposed mechanism, these studies [77–79] provide convincing evidence that m6A directly impacts heterochromatin formation. Furthermore, expanding evidence has revealed the role of m6A methylation on chromosome-associated regulatory RNAs (carRNAs) or mRNA-encoding histone-modifying enzymes and accessible chromatin. There is evidence of reverse feedback of histones on m6A modifications and of the histone elongation mark H3K36me3 guiding m6A deposition [80]. Kan et. Al more extensively discusses these concepts [73].

It is currently unknown if and how RNA modifications other than m6A feedback on epigenetic checkpoints.

### Viral mimicry

Viral mimicry describes an active anti-viral cellular state triggered by an endogenous stimulus. It may evoke innate and adaptive immune responses and can be triggered by cytosolic RNA or DNA [81].

Two landmark papers in 2015 describe “viral mimicry” as a process involving inhibition of epigenetic silencing, retrotransposon transcription, and IFN activation. The host cells interpret aberrant repetitive element RNA expression as a viral infection and activate an IFN response [82, 83]. Recognition of retrotransposon-derived duplex RNAs (dsRNA) by cytosolic RNA sensors like MDA5 or endosomal RNA sensors such as Toll-like receptor 3 (TLR3) initiates antiviral signaling. Stimulation of RNA sensors is propagated by mitochondrial antiviral-signaling protein (MAVS) aggregation on the mitochondrial surface, which induces a TBK1-mediated phosphorylation cascade that results in the phosphorylation, dimerization, and nuclear localization of IRF3/7 to activate either type I or III IFN signaling. This pro-inflammatory immune response ultimately suppresses proliferation and induces apoptosis in the affected cell. In addition, viral mimicry enhances adaptive immune responses as hERV-derived peptides form tumor-associated antigens (TAAs) that may elicit CD8 + T-cell responses. Glioblastoma is characteristically considered to be an “immunologically cold” tumor. Using adjunctive agents to enhance intratumoral viral mimicry and its innate and adaptive immune responses could bolster the effects of present immunotherapies.

In glioblastoma cell lines, DNA methyltransferase inhibition (DNMTi) increased the expression of TE and HERV-derived peptides [84]. However, clinical trials utilizing adjuvant DNMTi have been unsuccessful to date. A phase 1 trial using 5-Azacitidine monotherapy in recurrent high-grade IDHm gliomas reached disease stabilization in approximately 40% of patients but failed to accomplish a durable radiographic response [85]. Histone deacetylase inhibitors (HDACi) such as Vorinostat, which has a narrow therapeutic index, have shown safety/tolerability for the treatment of recurrent glioblastoma [86–88] but have not yet been combined with immunotherapies for clinical treatment of high-grade gliomas. In addition, the clinical efficacy of HDACi in improving progression-free or overall survival has yet to be demonstrated.

Several recurrent cancer-driving mutations are known to activate TEs that prime tumors for viral mimicry induction. One of the most studied examples is the H3.3^K27M^ mutation in high-grade gliomas [89]. This mutation impairs the recruitment of the Polycomb complex, reduces facultative heterochromatinization, and thus activates DNA transposons, LINEs, and SINEs [81]. However, despite elevated retrotransposon expression, H3.3^K27M^ gliomas do not have elevated IFN signaling, likely secondary to a cancer-specific compensatory mechanism. Treatment of high-grade glioma with either DNMTi or HDACi has been shown to more strongly promote MAVS-dependent induction of IFN and IFN-stimulated genes in H3.3K27M cancers than H3.3 wild-type cancers. The resulting enhanced dsRNA responses promote PKR-mediated cell death [89]. Therefore, although H3.3K27M gliomas lack IFN induction at baseline, H3K27me3 loss results in elevated retrotransposon expression and primes for viral mimicry responses to DNMTi and HDACi treatment.

Aside from ERVs, numerous studies have linked LINEs and SINEs to a viral mimicry response [12]. In fact, the cytosolic dsRNA sensor MDA5, a vital player in viral mimicry, was found to preferentially bind to the stem-loop structure formed by inverted-repeat Alus (IR-Alus) relative to the bidirectionally transcribed dsRNA structures of ERVs and LINEs [90]. IR-Alus is also the primary substrate for the A-to-I mRNA editor ADAR1 [91], which produces a modification that disrupts the RNA duplex and prevents MDA5-mediated dsRNA sensing [92]. Therefore, ADAR1 depletion increases cytosolic dsRNA levels and sensitizes cancer cells to treatment by viral mimicry inducers such as decitabine or CADK4/6 [90]. Furthermore, tumors with intrinsically high IFN signaling have been found to be ADAR1 dependent [93, 94], and thus, ADAR1 inhibition may be exploited for these viral mimicry-primed cells. On the other hand, immune-checkpoint blockade-resistant tumors in mice were found to be sensitive to both IFN and anti-PD1 blockade in ADAR1 deletion [95]. As immune checkpoint blockade resistance is common in high-grade gliomas, combined ADAR and epigenetic therapies may represent a novel path for a synthetic vulnerability to immunotherapies.

The mechanisms that dictate whether elevated dsRNAs induce sublethal or lethal IFN responses remain unclear. Survival of the subset of cancer cells with both high dsRNA levels and ADAR1 deficiency suggests that ADAR1-mediated editing is not the only mechanism cancer cells utilize to evade immunogenic cell death. As we strive to develop more robust and clinically relevant drugs, other RNA regulators and editors providing compensatory immunosuppression must also be explored. One recently found example is the RNA helicase DHX9 in breast cancer cells [96].

Long read methylome data indicates that hERV CpG methylation is lower at baseline than other TEs and the remaining genome in normal tissues [97]. Thus, it is probable that in many tumor cells, DNA methylation may not be the principal mechanism limiting ERV activation [98]. Though HDAC inhibitors and lysine methyltransferase inhibitors have been shown to synergize with DNMTis to activate ERVs [99, 100], other repressive and less characterized pathways likely provide compensatory effects. Further studies and improvements in epitranscriptomic mapping of gliomas are necessary to identify specific cancer-driving mutations in RNA modification regulators that prime tumors for viral mimicry. With crosstalk to epigenetic silencing already established, it is probable such mutations exist and may represent promising targets for novel high-grade glioma treatment in combination with immunotherapies and pre-transcriptional ERV regulation inhibitors (i.e., DNMTi and HDACi). Though several small molecule inhibitors of RNA modification regulators already exist, many must be redesigned to improve their BBB penetrance and levels in GBM. CRISPR-Cas systems developed for programmable RNA modification editing [101] and nucleotide-specific editing [102] expand the scope of RNA engineering and facilitate mechanistic understanding of the epitranscriptome. Like their DNA-targeting counterparts, RNA-targeting CRISPR-Cas systems may begin to be translated into the clinic in the coming years.

Post-transcriptional methods of viral mimicry induction are undergoing further study for recurrent cancers like glioblastoma. Despite its promise, the potential benefits of leveraging inducible hERV activity against solid tumors must be closely balanced with possible unintended consequences such as ERV onco-exaptation.

## Future directions & translational endeavors

Several drugs, such as 5-azacytidine and decitabine inhibiting DNA methylation, are FDA-approved for hematological tumors. Of the many similarities and parallels between DNA and RNA methylation, the most similar is m5A. Interestingly, studies show the clinical response to hypomethylating therapies do not correlate with DNA methylation status, and approximately 90% of 5-azacytidine is incorporated into RNA [103]. Thus, these therapies’ antiproliferative effects, either RNA- or DNA-mediated, and drug-repurposing potential remain hotly debated, with further mechanistic studies needed.

Though no FDA-approved drugs currently target epitranscriptomic regulators, there is preclinical evidence of the anti-tumor effects of RNA-modifying therapy and a growing cadre of interested biotech companies [103]. Most small molecule inhibitors for RNA-modifying therapy have been developed against m6A regulators. For example, the METTL3–METTL14 catalytic activity inhibitor STM2457 has been validated in vitro and in vivo for hematological tumors [9]. Additionally, inhibitors against FTO, ALKBH5, IGF2BP1, and ADAR1 have shown promising anti-tumor properties in vitro and in vivo [104]. However, as mentioned previously, these inhibitors will likely require chemical modification to improve their BBB penetration for glioma treatment.

Utilizing glioblastoma stem cells (GSCs) and xenograft mice, MA2, the ethyl ester form of meclofenamic acid (MA), an FDA-approved non-steroidal anti-inflammatory drug, was identified as a selective inhibitor of FTO that increases m6A mRNA levels and suppresses GSC-initiated tumor progression [105, 106]. Similarly, small-molecule compounds inhibiting pseudouridine synthase 7 impair GSC growth in vitro and, in mouse xenografts, improve prognosis [107]. Lastly, inhibition of TYK2, a mediator of both PUS7- and ADAR1-containing pathways in GSCs, demonstrated impaired GSC self-renewal and stemness [108]. As these molecules enter clinical trials, how they affect the landscape of cancer therapies targeting gene expression dysregulation and impact GBM patient outcomes will be exciting.

## Data Availability

Not applicable.
